# Toward Detecting the Zone of Elite Tennis Players Through Wearable Technology

**DOI:** 10.3389/fspor.2022.939641

**Published:** 2022-06-17

**Authors:** Hayati Havlucu, Baris Akgun, Terry Eskenazi, Aykut Coskun, Oguzhan Ozcan

**Affiliations:** ^1^User Experience Research Group (LUX), University of Lapland, Rovaniemi, Finland; ^2^Department of Computer Science, Koc University, Istanbul, Turkey; ^3^Department of Psychology, Koc University, Istanbul, Turkey; ^4^Koc University-Arcelik Research Center for Creative Industries (KUAR), Koc University, Istanbul, Turkey

**Keywords:** sports, psychological states, deep learning, flow state, machine learning

## Abstract

Wearable devices fall short in providing information other than physiological metrics despite athletes' demand for psychological feedback. To address this, we introduce a preliminary exploration to track psychological states of athletes based on commercial wearable devices, coach observations and machine learning. Our system collects Inertial Measuring Unit data from tennis players, while their coaches provide labels on their psychological states. A recurrent neural network is then trained to predict coach labels from sensor data. We test our approach by predicting being in the zone, a psychological state of optimal performance. We conduct two experimental games with two elite coaches and four professional players for evaluation. Our learned models achieve above 85% test accuracy, implying that our approach could be utilized to predict the zone at relatively low cost. Based on these findings, we discuss design implications and feasibility of this approach by contextualizing it in a real-life scenario.

## 1. Introduction

**Figure 1:**
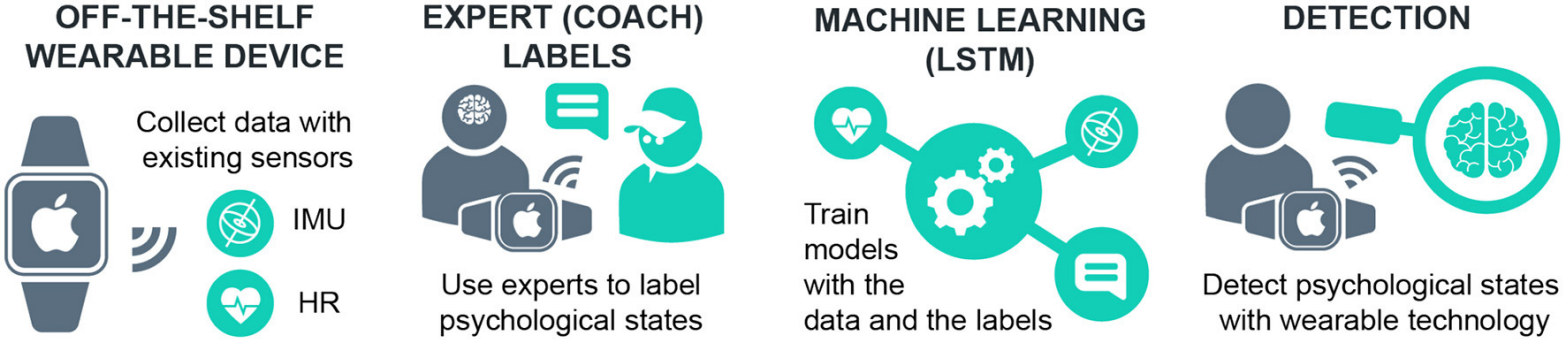
Summary of our approach to detect psychological states with wearable technology, expert labels and machine learning.

Research on sports wearable technologies suggest that athletes are not satisfied by raw physical data and demand feedback on their psychological states (Havlucu et al., 2017). However, the state of the art methods fall short in tracking the psychological states to provide feedback in realistic environments (Reinecke et al., 2011). Existing wearable devices offer affordability and convenience, but are limited to providing physiological information, such as heart rate (Aroganam et al., 2019). Considering both limitations, our motivation is to create affordable and convenient methods to track and give feedback on psychological states. We approach this motivation by correlating the physiological data collected from conventional wearable devices such as Apple Watch with labels from expert coaches, who are shown to observe psychological states in their players (Bakker et al., 2011; Havlucu et al., 2018), and training models to detect these states through machine learning (Figure 1).

We introduce a preliminary exploration to this approach by tracking what the coaches call being-in-the-zone (Young and Pain, 1999) in tennis. We picked tennis, since it is physically intense and mentally challenging (Fernandez et al., 2006). Based on the interview findings of previous research with tennis coaches, we focused on being in the zone as a psychological state (Havlucu et al., 2018). We conducted an experimental study involving two tennis games, with two elite coaches and four professional players. Our goal was to predict if tennis players are in the zone, by utilizing the Inertial Measuring Unit (IMU) data from Apple Watch Series 2 and coaches comments on their players zone. We used a deep recurrent neural network (LSTM) model to learn and was able to predict whether a tennis player is in the zone with around 85% test accuracy. Based on these results, we discuss the design implications and the feasibility of our approach. We also contextualize our approach in a real-life scenario.

The main contribution of this work is two-fold; (1) a novel step toward detecting psychological states through off-the-shelf wearable technology, machine learning and expert labels, and (2) design implications and the feasibility of such technology for HCI. Note that this work is a preliminary exploration for tackling our broad motivation, due to the extremely challenging nature of detecting psychological states and conducting longitudinal studies. We believe our findings can guide sports researchers and interaction designers on how to detect psychological states, as well as present particular directions for future of wearable technology.

## 2. Background

Tennis is among a few individual sports, which has both self-paced (serve) and externally-paced (ground strokes) performances with continuous switches intense physical effort, all of which trigger shifts in their mental states (Fernandez et al., 2006). Therefore, tennis players wish to get feedback on the mental aspects of their performance, rather than feedback on their physical performance (Havlucu et al., 2017). These mentally challenging features make tennis a great case for exploring the psychological measures of a sports performance.

Flow state, commonly known as 'the zone' for sports (Young and Pain, 1999), is a psychological state the state of optimal experience and performance (Jackson and Csikszentmihalyi, 1999). In the zone, athletes describe being immersed in and in total control of their performance effortlessly, which leads to their ideal performance (Kimiecik and Stein, 1992). Therefore, it is the state every athlete aspires get into. Research on the zone presents characteristics and dimensions to experience the zone (Jackson and Csikszentmihalyi, 1999). However, experiencing the zone consistently has been shown to be extremely challenging. Researchers have investigated the relationship between tennis performance and the zone (Koehn et al., 2013). Their findings indicate that the zone is a valuable psychological state to assess tennis performance. Studies on other sports such as football revealed coaches rating of players' performance included a significant correlation to their self-rated zone experience (Bakker et al., 2011). Specifically for tennis, these results were in line with the findings of Havlucu et al. that coaches could observe the zone of their players while rating their performance (Havlucu et al., 2018). They further elaborate that coaches track the body language, posture, activity and rituals of the players as the tennis specific cues of the zone.

## 3. Related Work

Current state-of-the-art fall short in tracking psychological states of athletes since measuring these states rely on invasive methods and can not be applied in real sports settings (Reinecke et al., 2011). The conventional method to measure psychological states is subjective scales (Jackson and Eklund, 2002), which include items rated by the participants and are administered through the Experience Sampling Method (ESM) (Csikszentmihalyi and Larson, 2014). ESM, Researchers probe participants at various intervals to fill out the scales. However, ESM intrudes into participants performance. In a sports setting, this intrusion can trigger unwelcome shifts in psychological states. Unlike ESM, our approach does not interfere with the participants activities during data collection.

Another approach is to correlate psychological states with psychophysiological measures. For example, researchers argue that heart rate variability (HRV) and respiratory rate are reliable indicators for different psychological states and use bulky and expensive equipment like electrocardiography and electroencephalography (EEG) to detect these states (Nacke and Lindley, 2008). Yet, we should note that the technology is advancing and these devices have become cheaper since the last decade. Thus, we see chest straps being utilized to measure HRV during matches in tennis (Fuentes-Garćıa et al., 2022) and racket sports (Parraca et al., 2022). Although, the same is true for measuring EEG with textile head ware (Pineda-Hernández, 2022), it still very challenging for athletes to perform a sports activity, especially professionally. These methods are still far from having the convenience and affordability of using current wearable technologies. In contrast, we use a machine learning approach to successfully predict the psychological state of 'zone' with wearables.

Human Activity Recognition (HAR) employs sensors from wearable devices and machine learning to predict and recognize diverse physical activities (Wang et al., 2019). In sports cases, multiple studies use IMU data to effectively recognize sports actions (e.g., forehand) (Connaghan et al., 2011). Although this approach is only used for detecting physical activity, the affordability and convenience proposed overcomes the limitations of the aforementioned methods. Therefore, we were inspired by HAR to create our approach to detect psychological states.

Psychological states manifest in behavioral responses along with physiological responses, which could be detected by HAR. Previous research illustrates with music, participants' walking style and rhythm change reflecting changing stress levels (psychological states), which is sensed and analyzed by IMUs attached to their heads (Tateyama et al., 2019). Tennis players also show behavioral responses while experiencing 'the zone'. Elite tennis coaches can observe the zone from their players' body language, posture, activity and rituals (Havlucu et al., 2018). They argue these cues are relevant to the movement and behavior of the players, which suggests IMUs could be used to detect these states.

## 4. Method

Our goal is to track the zone by utilizing data from off-the-shelf wearable technologies. We decided to limit the data to IMU for two reasons. First, tennis coaches observe the zone from their players movement and behavior, which can be quantified by IMUs (Havlucu et al., 2018). We were also inspired by research on other fields, for instance music, that report behavioral responses of psychological states such as distress levels could be successfully detected with IMUs (Tateyama et al., 2019). Second, IMU sensors are present in many off-the shelf wearable devices, unlike heart rate or EMG sensors. In our case, IMU offered affordability and convenience compared to other sensors, while providing high accuracy for our trained models (see Results).

### 4.1. Machine Learning Tasks

There are several questions of interest that we want to address with multiple machine learning tasks. The answers have design implications on the use cases of our approach:

*1) Can we detect the psychological state of being in the zone of a single or multiple tennis player(s) with a model learned from physical IMU data?* This question deals with the core idea of the paper; whether the movement information contained in the IMU data can be used to detect 'the zone'. For this, we define two machine learning tasks: (a) Use the data of each player separately and learn personalized models for each player. (b) Use the data of all the players which involves learning a single aggregate model from all the player data. We look at the test accuracies of both tasks to decide on the answer.

*2) Can a learned model generalize to new players?* This question deals with whether a learned model can predict the zone labels of a previously unseen player. If the answer is positive, a generalized model can be produced to detect 'the zone' of every player. If it is negative, we need to collect data for each player, i.e., the models need to be personalized. The associated machine learning task: (a) Use the data of 3 players for training and the data of the remaining for testing, and rotating the tested player. We look at the test accuracies to decide on the answer.

*3) If a learned model cannot generalize, can it be re-trained to speed up learning for a new player?* This question deals with if it is beneficial to re-use a learned model, if the answer to the previous question is negative or if the resulting performance is low. If the answer is positive, the data collection and training duration can be shortened. The associated machine learning task: (a) Re-train the models from the task 2a using the remaining players data. We compare the number of training epochs to reach an average test accuracy threshold (80%) between the models trained from scratch (in the task 1a) and the re-trained models, and testing accuracies to decide on the answer.

### 4.2. Machine Learning Formulation

We formulate the zone state detection as a binary classification problem where the windows of 12-dimensional IMU time series as the input and the latest coach label as the target. We use a Long Short Term Memory (LSTM) recurrent neural network model for all the ML tasks with a windows size of 50 (5 s). The structure of neural network includes two stacked LSTM layers with hidden unit sizes of 64 and 32 respectively, followed by a fully connected layer with 8 ReLU neurons and a single sigmoid output. We apply a dropout rate of 0.5 after the first LSTM layer. We use cross entropy loss, Adam optimizer with learning rate of 0.0025 and batch size of 128. Each model is trained for 100 epochs. The exact machine learning approach is of limited importance for our purposes. LSTM based models are widely used and well-established in activity recognition that are shown to be successful (Wang et al., 2019). LSTMs can successfully model the temporal nature of the data. Our model produced sufficient results for the sake of this study. However, we do not claim that it is the *best possible model*.

#### 4.2.1. Train-Test Splitting

**Figure 2:**
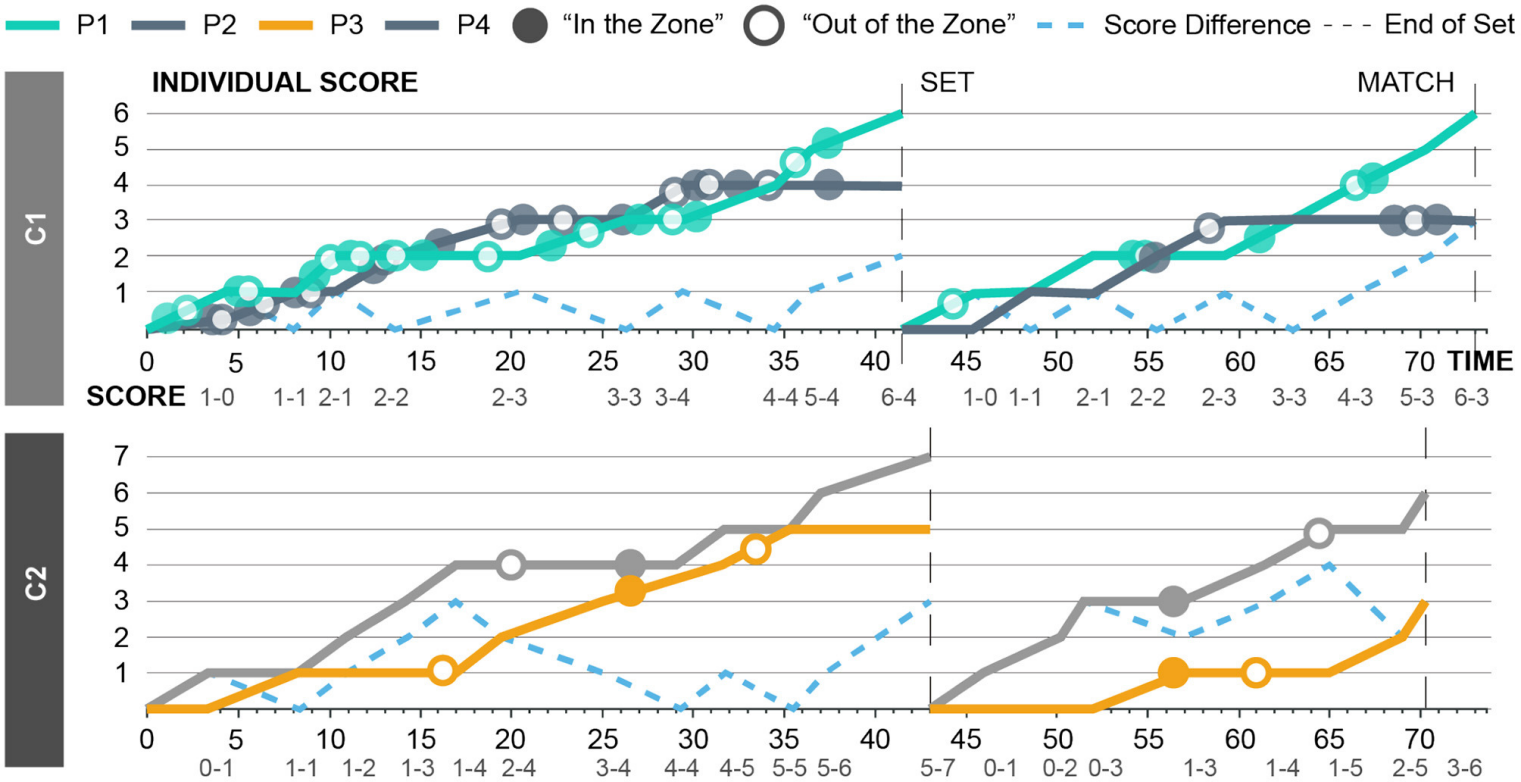
Labels of coaches, C1 (top) and C2 (bottom), for their respective players (P1 and P2, P3 and P4) according to individual scores and score differences.

The coach labels are not distributed uniformly in time (see Figure 2 in Results). This makes it difficult to perform standard time-series data splitting such as only taking the last 20% for testing. However, the label ratios are more or less 50%. Thus, we randomly pick parts from the data of at least 50 points and remove them as the test set. We then use the remaining data as the training set. This makes sure that the train and test sets have roughly the same ratio of labels and that no point in the test set is ever in any of the windows of the training set. The amount of data for the test set is picked to roughly give a 1/4 ratio of test data to train data. We perform this random splitting 5 times and report the average results.

### 4.3. Participants, Procedure, Setting, and the Data

Two elite level tennis coaches and four professional players participated in the experiment. The coaches had 25–28 years of tennis experience, with 9–11 years of professional coaching. They were both male, 35–38 years old, respectively. We described our aims to the coaches prior to conducting the experiment. Both coaches were knowledgeable of the zone from their own and their player's experience and shared they could observe the zone of their players. Accordingly, each coach was asked to select two of their professional players. All players were male, because the coaches only trained male players. Previous research demonstrated that gender has no significant effect on the zone in tennis (Koehn and Morris, 2012). The players were aged between 18 and 20 (*M* = 19.0, *SD* = 0.7), and all have participated in and won international tournaments.

We conducted the experiments on the coaches registered tennis club indoor hard courts. In each experiment, one coach's two players played a game, while their coach observed the players simultaneously. The coaches and the players were different in both games (Coach 1 with Player 1 and 2, Coach 2 with Player 3 and 4). The games were in best-of-three format and each lasted around 75 min. During the games, each player was asked to wear Apple Watch Series 2 on their dominant wrists, which was decided according to the arm players used for the racket. We collected 10 Hz IMU data from these devices. The IMU data, measured at each time step, is a 12 dimensional vector consisting of acceleration (3-dim), gravity vector (3-dim), orientation (3-dim) and rotation-rate (3-dim). Simultaneously, the coaches were instructed to label their players getting in and out of the zone according to their own observations of the players' movement and behavior. Due to their experience of the zone, the coaches were free to share and label any observation they have found relevant. These labels were used as binary data. The exact timing of these labels were stamped by the coaches. The content of the labels were written down by the experimenter and then matched with the timestamps. The total number of IMU measurements were about 193,000 with 47 labels by the first coach and 9 labels by the second. The duplicates were removed and the missing time stamps were filled with linear interpolation before learning.

## 5. Results

### 5.1. The Games and the Labels

Both games ended with two consecutively won sets. The first game was more intense and contained many comebacks. The coach from the first game (C1) labeled more instances for both players (P1 and P2, 47 vs. 7) (Figure 2) and the content of these labels were more elaborative (i.e., C1 - “[P1] is doing his rituals. His gaze is sharpened. He is controlling his breath.”). On the other hand, the coach from the second game (C2) only labeled the entrance to and exit from the zone. However, the LSTM model was only trained with binary labels (“In the Zone” and “Out of the Zone”).

### 5.2. The Machine Learning Tasks

To summarize, our evaluations answered the 1*st* and the 3*rd* questions positively, and the 2*nd* question negatively. All of our results are presented in Table 1. In this section, we elaborate on the results and explain the findings.

**Table 1:** The testing results of tasks explained in the Section 4.1 using 5 train-test splits and 100 epochs.

**ML**	**1a Individual**	**1b Aggregate**	**2a Generalization**	**3a 80%**	**3a Accuracy**
**task**	**models**	**model**		**threshold**	
C1 P1	85.69% (1.14%)	N/A	50.52% (1.38%)	19 vs. 14	88.31% (0.73%)
C1 P2	78.49% (1.20%)	N/A	51.81% (0.57%)	N/A vs. 24	85.64% (1.59%)
C2 P3	87.22% (1.59%)	N/A	49.74% (1.90%)	23 vs. 5	88.44% (0.79%)
C2 P4	85.79% (1.28%)	N/A	52.00% (1.66%)	17 vs. 4	88.64% (1.79%)
All/Avg.	84.30% (2.63%)	83.24% (0.55%)	51.02% (2.93%)	N/A	87.75% (2.62%)

“*C” represents the coaches and “P” represents the players. The last row provides the average of the average accuracies of individual models for tasks 1a, 2a, and 3a. The 3a Threshold column shows the training epoch when the average accuracy is consistently better than 85% (random start vs. transferred). The values in parenthesis show the standard deviations*.

1) Coaches zone labels can be detected from IMU data in tennis with high accuracy.

The 1a and 1b columns of Table 1 show that the testing accuracy of the models learned from individual player data and from the aggregated data are above or close to 85% other than the second player of the first coach. Previous studies in HAR present accuracies between 80 and 90% to yield in successful detection (Connaghan et al., 2011). Given the challenging nature of detecting psychological states, these results show that our approach can be utilized to detect the zone state of a player. We conclude that the answer to the first question is positive.

2) The models cannot be generalized between players.

The 2a column of Table 1 shows that the testing accuracy of the learned models are the same as random guess. This suggests that, at least with our data, the learned models can not generalize to a previously unseen player and individual training is needed for our approach to work. As such, we conclude that the answer to the second question is negative. This result may be due to the highly personalized nature of the zone and psychological states as discussed in Section 6.1.

3) Learning can be sped up by utilizing a previously learned model.

The 3a Threshold column of Table 1 shows that the models converge faster if we retrain a previous model as compared to training from random initialization. Furthermore, the re-trained models obtained better performance with the same number of epochs, with all models surpassing 85%. This suggests that previously collected data has some use even if the models are not generalizable between players. This also suggests that there could be a weak shared representation among the players. We conclude the answer to the third question is positive. This positive answer has implications on how a model should learn from data. The results suggest that the more data is collected and more models are learned, the faster the learning will be. Another point is that the existing models may decrease the amount of data needed to learn.

## 6. Discussion

Our goal is to track the zone by utilizing data from off-the-shelf wearable technologies. The results suggest that our approach could be utilized to predict the zone of a tennis player with above 85% accuracy. In this section, we discuss the feasibility of our approach through design implications of the results, contextualize it in a real life scenario to illustrate the potential, present the limitations of the current study and point out future research directions.

### 6.1. Personalized Psychological States

We can train machine learning models to detect what coaches call 'the zone' for individual players with high accuracy. Additionally, we can train a single model for multiple players. Yet, these models do not generalize to new players. Thus, players must be observed and labeled before predicting psychological state. This may not be a shortcoming of our approach, but simply the nature of psychological states (Young and Pain, 1999). Previous research discusses the zone as a highly personal state. Athletes have their own individual experience in the zone and some aspects of this experience may not be observed in others. According to coaches, unique player behaviors provide cues about their state (Havlucu et al., 2018). They need to know the player well to perceive these states. We could argue that user's model cannot be used to predict another user due to the highly personal nature of 'the zone'. Nonetheless, our results suggest that previously learned models can partially be used. The personal nature of these states is an open area for future research.

### 6.2. Labeling “The Zone”

Each coachs labeling of the zone was different. Although the data trained in the model was binary (In the Zone and Out of the Zone), the number of instances were significantly higher in the first experiment (47 vs. 9). We emphasized that the coaches should be experts and need to know the players well to properly assess the zone (Havlucu et al., 2018). This means each player should be labeled only by their own coach. These coaches could label the zone differently. Thus, the labels they produce is subjective. This challenges the reproducibility of the labels and generalizability of the models. On the other hand, it proposes a system that produces personalized models for each user and illustrates the possibility of predicting the zone of individual players. Therefore, we believe subjectivity of the labels is not a major issue for this work. In fact, subjectivity could be a strength of the approach, as the players might need a subjective assessment of their psychological states, which is not provided by current wearable devices (Havlucu et al., 2018). Additionally, the first game was more competitive, which may have resulted in more switches between the psychological states of the players and more coach labels. However, more labels do not necessarily mean more robust measurement. We do not yet know the optimal amount or time (i.e., after, during or before points) of the labels to reliably measure these states. In any case, we need to investigate the quantity and temporal dimensions by recruiting more coaches and comparing their labels with the trained models. We believe this will allow a more coherent and reliable way of measuring psychological states and possibly present a guideline on how to effectively label these instances, which could be addressed by future studies.

### 6.3. Contextualization of the Approach

**Figure 3:**
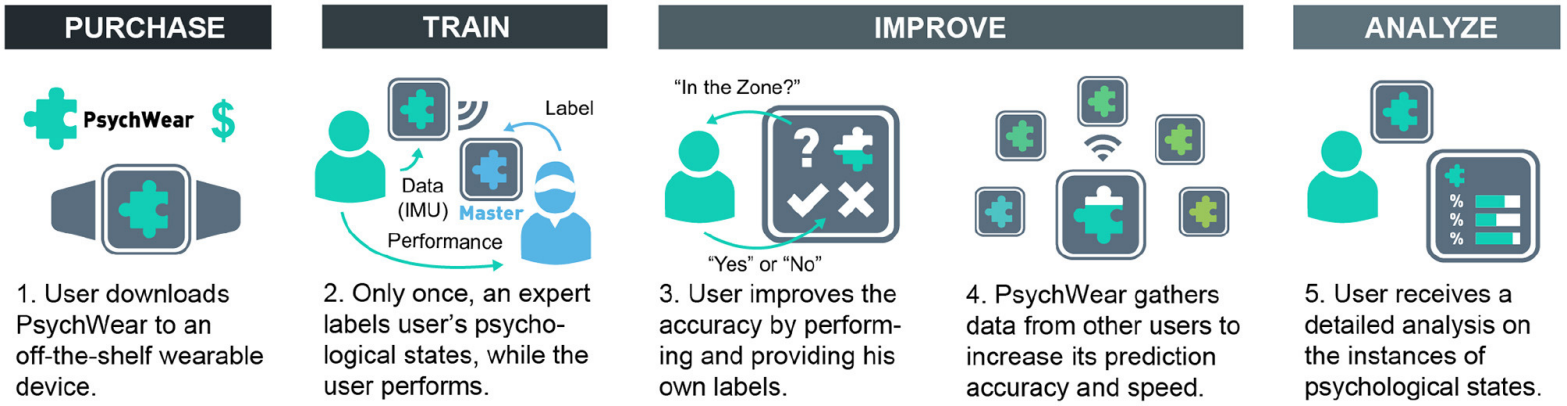
Contextualization of the approach: PsychWear's usage scenario and workflow.

Based on our findings, we envision that our approach can translate into a future wearable device application, PsychWear. Note that PsychWear does not aim to replace the coaches, but aims to supplement them. Figure 3 illustrates how PsychWear works. Tennis clubs buy affordable wearable devices and PsychWear for a small fee. Then the coaches provide labels for each player in training sessions. These sessions are required for each new player, because PsychWear can predict the zone specifically for each player (Section 6.1 and 2a column of Table 1). However, PsychWear can work with only one training session per player with high accuracy (1a and 1b columns of Table 1). During training, the players wear the devices and run PsychWear. They play a tennis game competing against other players, while PsychWear extracts the IMU data from the devices. Meanwhile, the coaches provide zone labels for each player (Section 6.2) using the master PsychWear application. PsychWear sends these data to the cloud, where the learning occurs, and then downloads the resulting model on the devices. This lets PsychWear increase its learning speed for new players (3a column of Table 1). Additionally, PsychWear can improve itself over every game. Players can choose to provide their own labels. This mode asks the players if they are in the zone upon prediction. The players provide a yes/no answer, which iterates PsychWears current data. Although this feature is not tested since the experiment is not repeated with the same participants, activity recognition literatur exhibits that improving the data set increases prediction accuracy (Wang et al., 2019). Moreover, PsychWear can be used in other physical activities in which the mental states of the performer has a significant effect on the performance outcome, and that have experts who can perceive the psychological states. Dancing, where the performance reflects the mental state, or yoga, where the mental processes are integrated to the physical activity are some examples. Exertion games, digital games that require physical effort, can also benefit from this tool.

### 6.4. Limitations

Although our results are promising to track the zone in tennis, the experiments were conducted with only two coaches and four players and were not repeated with the same players. The demanding nature of this experiment and its target group hindered rapid and repetitive measurement. We recognize our relatively small number of participants do not produce a generalizable and validated large-scale user study even with experts. Nonetheless, our aim in this study is to explore the novel concept of detecting psychological states through off-the shelf-wearable devices and machine learning, rather than validating the results. There are examples recently published in ACM conferences with similar number of participants. Khan et al. (2017) explored the feasibility of a novel and inexpensive activity recognition system in cricket with only 6 participants, who were mostly amateur players. Hölzemann and Van Laerhoven (2018) included only 3 participants for recognizing basketball activities with IMUs. We argue that our study contributes in exploring the feasibility of detecting psychological states with wearable technology. Note that, we have around 193,000 IMU measurements which by itself constitutes a large data set even with few participants. We plan to conduct more experiments and increase the participant pool. Moreover, we were not able to longitudinally evaluate the model. We need to know how well the model responds to previously trained players on different occasions. This will inform how many labeling instances are needed before accurately predicting the zone. Toward this end, we intend to repeat the experiments with the same players.

Additionally, we should mention limitations regarding our data collection. In each game, one coach was tasked to observe two players simultaneously, which may have challenged their concentration. Although, they are elite coaches, this may have resulted in data loss for observing both players. Moreover, the players were asked to wear the watches on their dominant wrists to collect more precise data on their movement with the racket. However, they were not used to wearing watches, thus their movement and the data collected might be hindered.

## 7. Conclusion

In this paper, we showed that wearable devices could help to detect psychological states. We introduced a novel approach that is utilized to predict 'the zone' of a tennis player with above 85% accuracy, by using Inertial Measuring Unit (IMU) data and elite coaches' labels on player performances. Primarily, we believe our exploration casts light upon how the future of wearable sports technology can detect psychological states of users. In broader terms, we argue this work presents novel directions for future wearable technology in HCI, and informs future studies aiming deeper understanding of the concept. However, the results should be treated within the discussed limitations, especially concerning the relatively small sample size. The nature of this work was to test the feasibility of the introduced approach. With this regard, we presented its limitations and pointed out design implications, rather than validating the results of the presented study. To this end, we contextualized this technical approach to a real life scenario that illustrates its potential. We believe that our contribution has thus become accessible for readers of non-technical backgrounds, namely interaction design and psychology researchers.

## Data Availability Statement

The raw data supporting the conclusions of this article will be made available by the authors, without undue reservation.

## Ethics Statement

The studies involving human participants were reviewed and approved by Koc University Ethical Committee. The patients/participants provided their written informed consent to participate in this study.

## Author Contributions

All authors listed have made a substantial, direct, and intellectual contribution to the work and approved it for publication.

## Conflict of Interest

The authors declare that the research was conducted in the absence of any commercial or financial relationships that could be construed as a potential conflict of interest.

## Publisher's Note

All claims expressed in this article are solely those of the authors and do not necessarily represent those of their affiliated organizations, or those of the publisher, the editors and the reviewers. Any product that may be evaluated in this article, or claim that may be made by its manufacturer, is not guaranteed or endorsed by the publisher.
